# Vitamin A Promotes the Repair of Mice Skeletal Muscle Injury through RARα

**DOI:** 10.3390/nu15173674

**Published:** 2023-08-22

**Authors:** Wenjia Zhang, Qingyun Peng, Xiaoyu Zhang, Jiaxu Guo, Huili Tong, Shuang Li

**Affiliations:** 1 Key Laboratory of Animal Cellular and Genetics Engineering of Heilongjiang Province, Northeast Agricultural University, Harbin 150030, China; zwj6270@163.com (W.Z.); 18937666889@163.com (Q.P.); zxy981762364@163.com (X.Z.); 13100919242@163.com (J.G.); tonghuili@neau.edu.cn (H.T.); 2 Laboratory of Cell and Developmental Biology, Northeast Agricultural University, Harbin 150030, China

**Keywords:** muscle injury repair, retinoic acid, RARα

## Abstract

Vitamin A (VitA) is an important fat-soluble vitamin which plays an important role in cell growth and individual development. However, the effect of VitA on the repair process of muscle injury and its molecular mechanism are still unclear. In this study, VitA and RA were first added to the culture medium of differentiated cells. We then detected cell differentiation marker proteins and myotube fusion. Moreover, the effects of VitA on RARα expression and nuclear translocation were further examined. The results showed that VitA significantly promoted the differentiation of C2C12, and the expression of RARα was significantly increased. Furthermore, VitA was injected into skeletal muscle injury in mice. HE staining and Western Blot results showed that VitA could significantly accelerate the repair of skeletal muscle injury and VitA increase the expression of RARα in mice. This study provides a theoretical basis for elucidating the regulation mechanism of VitA-mediated muscle development and the development of therapeutic drugs for muscle diseases in animals.

## 1. Introduction

In skeletal muscle, muscle satellite cells (SA) reside between the muscle cell membrane and the basement membrane and are a group of muscle-derived stem cells with the potential for both proliferation and differentiation [1]. Under normal circumstances, SA are in a resting state. When muscle damage, exercise or nutritional stimulation occurs, Myogenic regulatory factors (MRFs) are activated, SA proliferate and differentiate into muscle fibers, or fuse with the original muscle fibers to form multinucleated cell bodies, thereby promoting muscle development and regeneration. The MRF family consists of MyoD, MyoG, Myf5 and Myf6. Among these, MyoD and Myf5 play a major role in the formation of myoblasts, and their expression initiates the differentiation of muscle satellite cells into myoblasts [2]. MyoG and Myf6 play a major role in the terminal stage of myoblast differentiation, which can induce myoblast fusion and form myotubes [3]. In addition, desmin is a component of the intermediate filament in the cytoskeleton of muscle cells, which is involved in the support and movement of cells. It exists in the form of reticular structure in muscle tissue and is one of the marker factors of muscle differentiation.

Retionl (Vitamin A, VitA) stands as a pivotal nutrient with a multifaceted presence. It mainly exists in the retina of animal liver, bloodstream and eyeball, and can regulate a variety of development and metabolic processes in vivo [4,5,6]. When vitamin A is deficient, the body is in a state of immunodeficiency [7], which impairs innate immunity by hindering the regeneration of the mucosal barrier at the site of injury and reducing the function of neutrophils, macrophages and natural killer cells [8]. Vitamin A and its derivatives also play an important role in eye development and daily visual function. In the body, they trigger cascade reactions through metabolic cycles, regenerate visual chromophores to receive light, stimulate photoreceptors, and activate RPE cells, thereby affecting visual function [9]. In the reproductive process, vitamin A is involved in the initiation of meiosis in the female gonads of the embryonic period, and is involved in the signaling mechanism of initiating meiosis in the male gonads after birth. It is also essential for female mammary gland development and lactation yield [10].

Upon entering cells, Vitamin A undergoes a catalytic transformation via retinol dehydrogenase and alcohol dehydrogenase, culminating in the production of retinaldehyde (RAL). This intermediate is subsequently metabolized by retinol dehydrogenase into retinoic acid (RA). Functionally, RA predominantly associates with nuclear receptors, namely the retinoic acid receptor (RAR) and the retinoid X receptor (RXR). This binding event triggers gene transcriptional activity and activates downstream signaling pathways, thereby orchestrating cellular proliferation, differentiation, and the intricate regulation of tissue and organ formation [11]. RAR is encoded by three closely related genes located on different chromosomes, and the three proteins expressed are named α, β and γ, respectively. The incorporation of retinoic acid into the ligand-binding domain leads to conformational changes, thereby forming RAR homodimers or RAR/RXR heterodimers. The activated RAR homodimers recruit co-activators with histone acetylase activity, thereby opening the chromatin structure and achieving downstream transcription of regulatory genes [12]. Previous studies have reported that, in early promyelocytic leukemia cell lines, granulocyte differentiation is directly mediated by nuclear receptor RARα [13]. In rodents, administration of selective retinoic acid receptor γ agonists can improve neuromuscular strength in volume muscle loss models [14]. Furthermore, RARγ signaling was identified to inhibit the adipogenic differentiation of mesenchymal stromal cells [15]. This effect may also be part of the mechanism of improving muscle repair quality. However, the effect of a single RAR subtype on myogenesis has not been elucidated.

Several studies have demonstrated the effect of VitA on muscle development. It has been observed that Vitamin A can distinctively impede limb bud cell chondrogenesis while stimulating myogenesis [16]. Previous studies on Black Angus calves have shown that VitA injection to newborn calves can up-regulate the expression of myogenic genes (Pax3, Pax7, Myf5, MyoD) and myogenic proteins, thereby promoting calf muscle development [17]. Providing vitamin A to cattle at birth can increase carcass marble patterns without affecting animal body performance [18]. In addition, the metabolite of VitA, RA, is also a catalyst for myogenic differentiation [19]. Notably, in zebrafish, RA activates muscle differentiation in vivo through fibroblast growth factor 8 (Fgf8) gene signaling (a muscle differentiation activator) [20]. In sheep primary myoblasts, VitA inhibits cell proliferation by reducing cyclin D1 protein expression. However, RA can promote myogenic protein gene expression and MyHC protein levels, and up-regulate glucose transporter 4 (GLUT4) mRNA and protein expression [21].

Although studies have shown that VitA has a certain effect on muscle differentiation, its potential mechanism has not been elucidated. Therefore, based on the mouse C2C12 cell and skeletal muscle injury repair model, this study examined the effect and mechanism of VitA on the differentiation of mouse C2C12 cells, and clarified the effect of VitA on muscle repair after injury and the optimal concentration. The results showed that exogenous addition of 10 μM VitA or RA significantly promoted the differentiation of C2C12 cells, and the expression and nuclear translocation of RARα were significantly increased after VitA addition. In addition, intraperitoneal injection of 0.1 mg/kg VitA promoted the repair of skeletal muscle injury in mice. This study clarified the mechanism of VitA promoting skeletal muscle regeneration and has potential application value.

## 2. Materials and Methods

### 2.1. Reagents

ICR mice were purchased from Jilin Changchun YISI Laboratory Animal Technology Company. Mouse C2C12 cells were purchased from Procell (Wuhan, China). Retinol (VitA, R7632, purity ≥ 98%) and Retinoic acid (RA, R2625, purity ≥ 95%) were purchased from Sigma (Kawasaki, Japan). Dimethyl sulfoxide (DMSO, ab146588, purity > 98%) was purchased from abcom (Waltham, MA, USA). VitA powder was dissolved in dimethyl sulfoxide DMSO and diluted to different concentrations to treat C2C12 cell. Fetal bovine serum (FBS002), horse serum (YT2515), penicillin and streptomycin (YT2515) were purchased from Biological Industries (Cromwell, CT, USA). High glucose DMEM medium (11965092) was purchased from Gibco (Canberra, Australia). RIPA lysis buffer (P0013C) and DAPI (C1005) were purchased from Beyotime (Shanghai, China). Hematoxylin-eosin staining kit (G1120) was purchased from Solarbio (Beijing, China). Anti-GAPDH (sc-365062), Anti-MyoG (sc-52903) and Anti-MyoD (sc-377460) were purchased from Santa Cruz Biotechnology (Dallas, TX, USA), and Anti-RARα (A0370) was purchased from ABclonal (Wuhan, China). Goat anti-Rabbit Anti-IgG (A0208) was purchased from Beyotime (Shanghai, China).

### 2.2. Cell Culture

C2C12 cells were rapidly thawed in a 37 °C water bath, resuspended in 10% fetal bovine serum basic medium, and centrifuged at 1500 rpm for 5 min. Cells were resuspended in a 10% proliferation medium and seeded at an appropriate density in a culture container at 37 °C in a 5% CO_2_ incubator. C2C12 cells were cultured in DMEM containing 10% FBS. When the cells proliferated to a density of about 80%, 2% differentiation medium (DMEM containing 2% HS, 0.05 g/L penicillin, 0.06 g/L streptomycin and 3.7 g/L NaHCO_3_) was used to induce C2C12 cell differentiation. At the same time, the control group was added with 2 μL DMSO, and the experimental group was added with 2 μL vitamin A. The differentiation medium was changed every 48 h. The myotube fusion during cell differentiation was observed under an optical microscope.

### 2.3. Western Blotting

The protein was extracted from C2C12 cells or tibialis anterior muscle using a protein lysis buffer (RIPA), added to a 5× loading buffer at a ratio of 4:1, and then boiled in boiling water for 10 min. The target protein was separated using SDS-PAGE, transferred to PVDF membrane, and blocked with 5% milk blocking solution for 1 h. It was washed three times with distilled water for 5 min each time, and the primary antibody was added and incubated overnight at 4 °C. It was then washed four times with PBST for 8 min each time, the secondary antibody was added and incubated at 37 °C for 1 h. After PBST washing, the chromogenic solution was added to the chemiluminescence instrument for development.

### 2.4. Immunofluorescence and Ultra-High Resolution Microscopy

C2C12 cells were washed with PBS and fixed with 4% paraformaldehyde for 20 min. After washing with PBS, the cells were permeabilized with 0.5% TritonX-100 (dissolved in PBS buffer) for 20 min, and blocked with PBST containing 5% bovine serum albumin at 37 °C for 60 min. After discarding the blocking solution, the cells were incubated with the Desmin (1:50) or RARα (1:50) antibodies overnight at 4 °C. The cells were then incubated with goat anti-rabbits IgG/fluorescein isothiocyanate (FITC) (1:50) and donkey anti-rabbits IgG/ RBFITC (1:50) at 37 °C for 1 h. Finally, the slides were sealed with anti-fluorescence quencher and observed under fluorescence microscope (Olympus, BX43, Tokyo, Japan) and ultra-high-resolution microscope (Deltavision OMX SR, GE).

### 2.5. Animal Experiment

Animal experiments were approved by the Animal Protection Committee of Northeast Agricultural University (Animal Ethics Approval Reference No: NEAUEC2021 01 26) and carried out according to the “Chinese Animal Science Standards“ of the National Standardization Technical Committee of Animal Science.

Four-week-old female ICR mice were purchased from YISI Technology (Changchun, China). Mice were grown in a 12–21 h artificial simulated day and night cycle with adequate food and water supply. The muscle injury repair model was established in mice with a body weight of about 25 g, and the drug injection experiment of muscle injury was carried out. Mice were randomly assigned to two groups with 12 animals per group: control group, fed with standard diet; VitA treatment group, with intraperitoneal injection of VitA. After 3 days of treatment, 30 μL of 5% bupivacaine hydrochloride (BH) was injected into the tibialis anterior muscle (TA) of the mice’s legs to establish a skeletal muscle injury model. The tibialis anterior muscles of mice were isolated on days 0 (0 D), 1 (1 D), 3 (3 D), 5 (5 D), 7 (7 D) and 14 (14 D) after injury, and Western blotting or paraffin sections were performed.

### 2.6. Hematoxylin and Eosin Staining

The isolated tibialis anterior muscle (TA) was fixed in 4% paraformaldehyde/PBS for 24 h, dehydrated with gradient alcohol, embedded in paraffin, and cut into 7 μm thick sections. Then, HE staining was performed using a commercial kit (Solarbio, Beijing, China). After a short wash with tap water, neutral gum was used to seal the film and photographed under a microscope.

### 2.7. Statistical Analysis

Image J was used to scan the results of Western blotting and count the number of nucleus in the myotubes. The scanned data were analyzed using GraphPad Prism 7, and three independent repeated experiments were performed in each group of Western blotting. The data were analyzed with a T test. The experimental data were expressed as mean ± S.D. ns: not significant, * *p* < 0.05, ** *p* < 0.01, *** *p* < 0.001.

## 3. Results

### 3.1. Effects of VitA on Differentiation of C2C12 Cell

**Figure 1:**
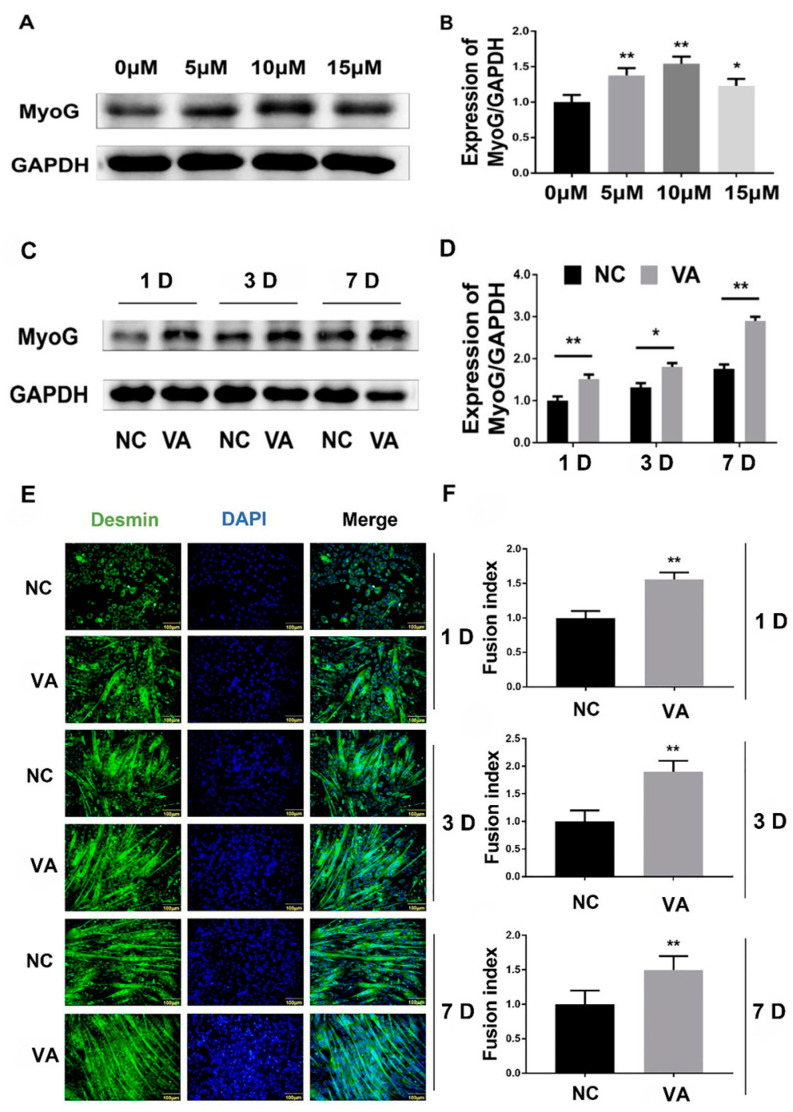
VitA affects on the differentiation of C2C12 cells. (**A**) After different doses of VitA induced C2C12 cell differentiation, Western blotting was used to detect the expression of MyoG protein. (**B**) gray scanning of MyoG protein in (**A**). (**C**) Exogenous VitA was added and C2C12 cells were induced to differentiate into 1 D, 3 D and 7 D, respectively. Western blotting was used to detect the protein expression of muscle differentiation marker molecule MyoG. (**D**) Gray scanning of MyoG protein in (**C**). (**E**) Exogenous addition of VitA and induction of C2C12 cell differentiation 1 D, 3 D and 7 D, respectively, immunofluorescence detection of Desmin. (**F**) quantitative analysis of the myotube fusion index expressed by Desmin in (**E**) statistics. The experiment was repeated three times.”*”: *p* < 0.05, “**”: *p* < 0.01. Scale bar = 100 μm.

C2C12 cells were treated with different concentrations of VitA (5 μM, 10 μM, 15 μM), and the culture medium was changed every 48 h. The cells were induced to differentiate into a large number of myotubes at 72 h. Western blotting results showed that the expression level of MyoG gradually increased with the increase in VitA concentration. The expression level of MyoG reached the highest when 10 μM VitA was added. It can be seen that VitA can promote the differentiation of C2C12 cells and the optimal concentration was 10 μM (Figure 1A,B).

In order to clarify the temporal effect of VitA on the differentiation of C2C12 cell, C2C12 cells were treated with an optimal concentration of 10 μM VitA, and cell protein samples were extracted at different stages of differentiation (1 D, 3 D, 7 D). Western blotting results showed that the expression of MyoG in the VitA treatment group was significantly higher than that in the control group at different differentiation stages (Figure 1C,D). The results of Desmin immunofluorescence showed that the myotube fusion rate of C2C12 cells treated with 10 μM VitA was significantly higher than that of the corresponding control group at 1 D, 3 D and 7 D of differentiation (Figure 1E,F), indicating that VitA promotes the differentiation of C2C12 cells.

### 3.2. Effects of RA on Differentiation of C2C12 Cell

**Figure 2:**
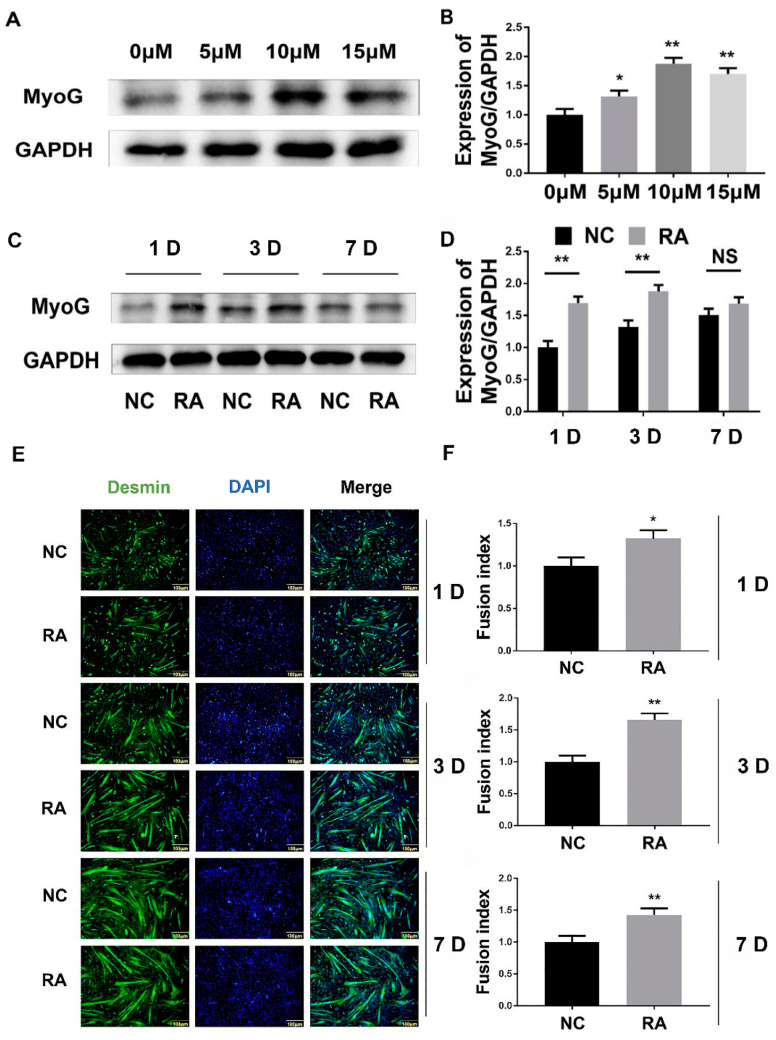
RA affects on the differentiation of C2C12 cells. (**A**) After different doses of RA induced C2C12 cell differentiation, Western blotting was used to detect the expression of MyoG protein. (**B**) Gray scanning of MyoG protein in (**A**). (**C**) Exogenous RA was added and C2C12 cells were induced to differentiate into 1 D, 3 D and 7 D, respectively. Western blotting was used to detect the protein expression of muscle differentiation marker molecule MyoG. (**D**) Gray scanning of MyoG protein in (**C**). (**E**) Exogenous addition of RA and induction of C2C12 cell differentiation at 1 D, 3 D and 7 D, respectively, and immunofluorescence detection of Desmin. (**F**) quantitative analysis of the myotube fusion index expressed by Desmin in (**E**) statistics. The experiment was repeated three times. “*”: *p* < 0.05, “**”: *p* < 0.01. Scale bar = 100 μm.

C2C12 cells were treated with different concentrations of RA (5 μM, 10 μM, 15 μM), and the culture medium was changed every 48 h. The cells were induced to differentiate into a large number of myotubes at 72 h. Western blotting results showed that the expression level of MyoG gradually increased with the increase in RA concentration, and the expression level of MyoG reached the highest at 10 μM. It can be seen that RA can promote the differentiation of C2C12 cells and the optimal concentration was 10 μM (Figure 2A,B). In order to clarify the time effect of RA on the differentiation of C2C12 cells, C2C12 cells were treated with RA at an optimal concentration of 10 μM, and cell protein samples were extracted at different stages of differentiation (1 D, 3 D, 7 D). Western blotting results showed that the expression of MyoG in the RA treatment group was significantly higher than that in the control group at different differentiation stages (Figure 2C,D). The results of Desmin immunofluorescence showed that the myotube fusion rate of C2C12 cells treated with 10 μM RA was significantly higher than that of the corresponding control group at 1 d, 3 d and 7 d of differentiation (Figure 2E,F), indicating that RA could promote the differentiation of C2C12 cells.

### 3.3. The Localization and Protein Expression of RARα during Differentiation after VitA Treatment

**Figure 3:**
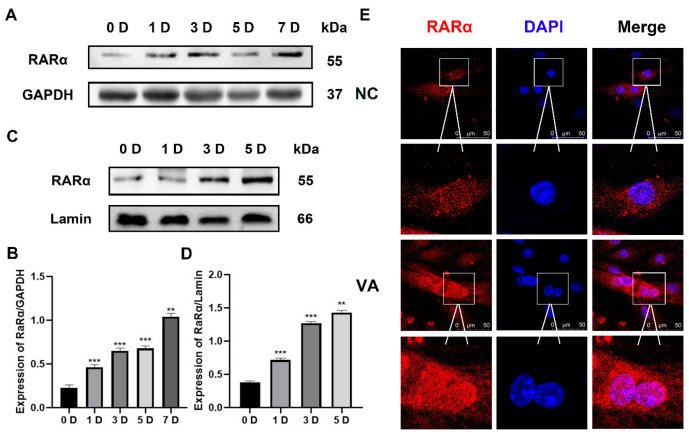
VitA affects the expression and the localization of RARα. (**A**) Exogenous VitA was added and C2C12 cells were induced to differentiate into 1 D, 3 D, 5 D, and 7 D, and the total protein of the cells was extracted. The protein expression of retinoic acid receptor RARα was detected with Western blotting. (**B**) Gray scanning of MyoG protein in (**A**). (**C**) Exogenously added VitA and induced C2C12 cells to differentiate 1 D, 3 D, and 5 D, respectively, and extracted nuclear proteins. The protein expression of retinoic acid receptor RARα was detected with Western blotting. (**D**) Gray scan of RARα protein in (**C**). (**E**) The localization of RARα in differentiated cells after VitA treatment. Red is RARα, blue is the nucleus. The experiment was repeated three times. “**”: *p* < 0.01. “***”: *p* < 0.001. Scale bar = 50 μm.

In order to explore whether VitA promotes C2C12 cell differentiation through RARα, C2C12 cells were treated with 10μM VitA, and the culture medium was changed every 48 h to induce cell differentiation. The total protein was extracted at 0 D, 1 D, 3 D, 5 D and 7 D of cell differentiation, and the protein expression level of RARα was detected using Western blotting. The results showed that VitA significantly up-regulated the expression of RARα at different stages of cell differentiation, and the expression of RARα gradually increased with the prolongation of cell differentiation time (Figure 3A,B). At the same time, the expression of RARα in the nucleus was further detected. The nuclear protein was extracted at 0 D, 1 D, 3 D and 5 D of cell differentiation, and the nuclear protein expression level of RARα was detected using Western blotting. The results showed that the expression of RARα gradually increased with the prolongation of cell differentiation time (Figure 3C,D).

Next, immunofluorescence staining of RARα was performed in this study, and the expression and localization of the two were observed with ultra-high resolution microscopy. When the cells were induced to differentiate to 72 h, RARα was mainly distributed in the cytoplasm in the control group, while RARα was mainly distributed in the nucleus in the VitA treatment group. It is indicated that VitA can promote RARα to enter the nucleus and accumulate in the nucleus (Figure 3E), suggesting that RARα is involved in and regulates the differentiation of C2C12 cell.

### 3.4. Effects of Different Doses of VitA on the Expression of Differentiation-Related Proteins in Mice

**Figure 4:**
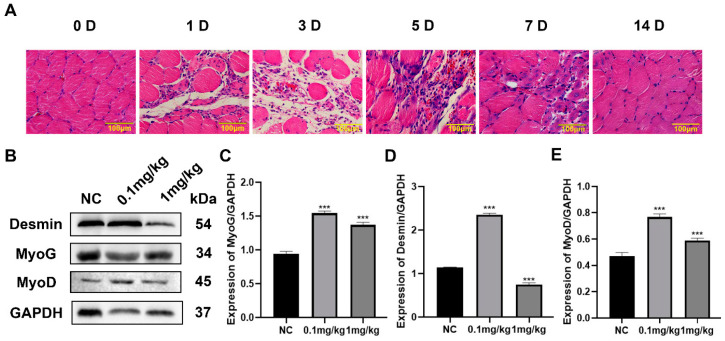
The optimal concentration of VitA to promote the repair of skeletal muscle injury in mice. (**A**) HE staining results of mouse muscle injury repair process. Red is the cytoplasm after eosin staining, and blue is the nucleus after hematoxylin staining. (**B**) Different doses of VitA were injected into mice after muscle injury, and the protein expression levels of MyoG, MyoD and Desmin were detected with Western blotting. (**C**) Gray scanning of MyoG protein in (**B**). (**D**) Gray scanning of Desmin protein in (**B**). (**E**) Gray scanning of MyoD protein in (**B**). The experiment was repeated three times. “***”: *p* < 0.001. Scale bar = 100 μm.

A muscle injury model was established by injecting 30 μL of 5% bupiVitAcaine hydrochloride (BH) into the anterior tibial muscle (TA) of both legs of mice. The tibialis anterior muscle of mice was isolated at 0 D, 1 D, 3 D, 5 D, 7 D and 14 D after injury, and HE staining was performed. The results showed that new muscle fibers began to appear on the 5th day after muscle injury in mice (Figure 4A), indicating that a large number of muscle satellite cells differentiated at this stage to promote muscle damage repair. Therefore, on the 3rd day before modeling, mice in the experimental group were intraperitoneally injected with 0.1mg/kg and 1 mg/kg VitA, respectively. On the 5th day after muscle injury, the tissue protein of tibialis anterior muscle was extracted, and the expression of muscle satellite cell differentiation marker protein was detected with Western blotting to determine the optimal injection dose of VitA.

Western blotting results showed that the expression levels of muscle satellite cell differentiation marker proteins MyoD, MyoG and Desmin after 0.1 mg/kg VitA treatment were significantly higher than those in the control group and 1 mg/kg VitA treatment group (Figure 4B–E), suggesting that 0.1 mg/kg VitA can be used as the optimal dose for subsequent muscle injury repair.

### 3.5. Effect of VitA on Muscle Injury Repair in Mice

**Figure 5:**
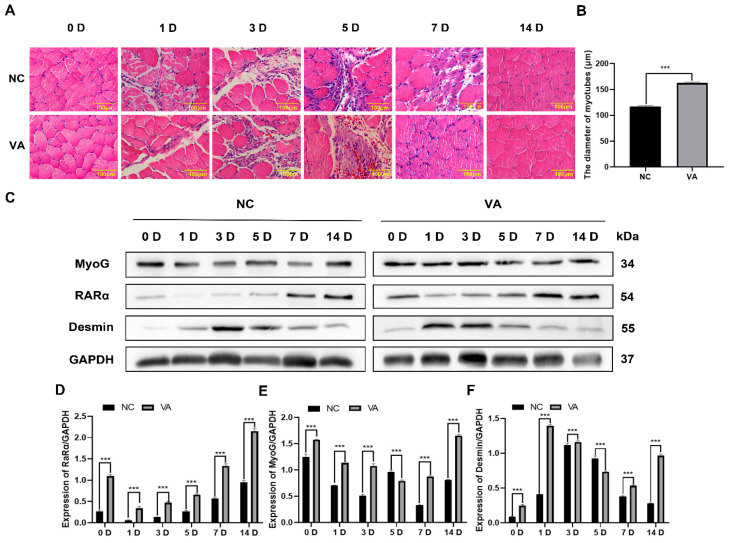
VitA’s effects on muscle injury repair in mice. (**A**) Mouse muscle injury repair process of tissue HE staining results. Red is the cytoplasm after eosin staining, and blue is the nucleus after hematoxylin staining. (**B**) Statistics of myotube diameter on the 14th day after injury. (**C**) After muscle injury in mice, 0 D, 1 D, 3 D, 5 D, 7 D, and 14 D tibialis anterior muscle tissue proteins were extracted, and the protein expression levels of muscle differentiation marker molecules MyoG, Desmin and RARα were detected with Western blotting. (**D**) Quantitative statistical histogram of RARα protein in (**C**). (**E**) Quantitative statistical histogram of MyoG protein in (**C**). (**F**) Quantitative statistical histogram of Desmin protein in (**C**). “***”: *p* < 0.001. Scale bar = 100 μm.

In order to clarify the effect of VitA on the repair of skeletal muscle injury in mice, the anterior tibial muscle of mice was injected at a dose of 0.1 mg/kg before injury, and HE staining was performed. The results showed that muscle fibers began to dissolve at 1 D after muscle injury in mice, and the degree of muscle injury in the VitA group was weaker than that in the control group, indicating that VitA intake could alleviate bupivacaine-induced muscle injury in mice. The dissolution of muscle fibers was aggravated at 3 D after injury, indicating that the muscle injury was serious. At 5 D, new muscle bundles were formed at the site of muscle injury. At 7 D after injury, it can be clearly observed that the diameter and number of new muscle fibers in the VitA treatment group were significantly higher than those in the control group, and the central nucleus appeared in the newly formed myotubes, indicating that the muscles were completely restored. In the control group, the muscles were completely repaired at 14 D after injury (Figure 5A). The diameter of newly formed muscle fibers in the VitA treatment group was significantly higher than that in the control group (Figure 5B).

In this study, Western blotting was used to detect the expression of muscle satellite cell differentiation marker protein and retinoic acid receptor protein during injury repair. The results showed that the expression of MyoG and Desmin was gradually up-regulated during injury repair, and the expression levels of MyoG and Desmin in the VitA treatment group were significantly higher than those in the control group, suggesting that VitA can promote the differentiation of muscle satellite cells. At the same time, the expression of RARα in the VitA treatment group was always higher than that in the control group (Figure 5C–F), which was consistent with the results of in vitro experiments, indicating that VitA can be metabolized into RA in vivo and bind to its receptor, thereby promoting muscle damage repair.

## 4. Discussion

Skeletal muscle, as the largest tissue of the body, represents approximately 40% of adult animals’ body weight and accounts for 50–75% of the total body protein [22]. It plays a regulatory role in exercise, metabolism and overall homeostasis. VitA is an important nutrient in the body. VitA and its metabolite RA play a vital role in muscle development. Studies have shown that RA can actiVitAte RXR and RAR signaling to promote myogenic differentiation of C2C12 cells and human skeletal muscle primary satellite cells [23,24]. Although some studies support that VitA and its metabolites have a positive effect on muscle health, their effects on muscle injury repair are still unclear. We focused on the role of VitA in the repair of muscle injury and tried to reveal its potential mechanism. The results of this study show that VitA can up-regulate the expression of RARα and promote its entry into the nucleus, accelerate the repair process of muscle injury in mice, and promote the differentiation of C2C12 cells.

In this study, C2C12 cells cultured in vitro were induced to differentiation into myotubes by adding different concentrations of VitA and RA. After 1 D, 3 D and 7 D of differentiation, Western blotting results showed that the expression level of MyoG in the experimental group was significantly higher than that in the control group. Immunofluorescence staining showed that the fusion rate of myotubes after adding VitA and RA was higher than that of the control group. This indicates that VitA and its metabolite RA can promote the differentiation of C2C12 cell, but its potential mechanism has not been elucidated. Previous studies have shown that vitamin A can promote the differentiation of mouse C2C12 cells through the formation of retinoic acid by DHRS3 [25], which is consistent with the results of this study.

RAR can mediate the active metabolite RA of VitA [26,27,28]. RAR forms a heterodimer with RXR, binds to the RA response element in the promoter region of the target gene, and activates gene transcription when the ligand binds [29,30,31]. Therefore, in this study, the total protein and nuclear protein were extracted, and the expression of RARα was detected to clarify the pathway of VitA affecting the differentiation of C2C12 cell. Western blotting results showed that the expression of RARα gradually increased with cell differentiation.

Since RARα is mainly distributed in the nucleus, the localization of RARα during C2C12 cell differentiation was determined through ultra-high-resolution microscopy. In differentiated C2C12 cells, the nuclear translocation of RARα in the VitA treatment group was significantly higher than that in control group, indicating that VitA promoted the nuclear translocation of RARα. The results of this study indicate that VitA promotes C2C12 cell differentiation by metabolizing to RA and binding to its nuclear receptor RARα. Previous studies have shown that overexpression of MyoD can activate RA-induced myogenesis (i.e., RA differentiation and anti-proliferation) and induce the expression of RXRα gene [32,33,34]. In muscle cells, the RXR-MyoD complex is recruited at the DNA binding site of MyoD. RAR can also be used as a transcription factor to initiate the dynamic changes of chromatin in RA reaction sites by recruiting chromatin modifying enzymes and p300 [35,36]. The interaction between RXRα and muscle basic helix-loop-helix (b-HLH) proteins occurs through the binding of muscle b-HLH protein subunits to the DNA domain of RXRα [37]. It has been speculated that there is also a special relationship between RARα and muscle differentiation markers. One is the interaction between RARα and muscle differentiation marker factors. Another is that RARα may act as a transcription factor to directly or indirectly regulate the expression of muscle differentiation marker genes. Therefore, our future research will focus on the interaction between RARα and differentiation marker factors such as Desmin, MyoD and MyoG, and further explore the pathway of RARα affecting cell differentiation.

In order to further verify the effect of VitA on muscle development, this study established a mouse muscle injury repair model. Zhao et al. have shown that supplementation of RA to obese mice can not only promote its proliferation in the early stage of muscle regeneration, but also promote apoptosis in the remodeling stage of muscle regeneration, which is beneficial to muscle regeneration damaged by obesity [38]. Di et al. showed that RARγ agonists can promote the repair of various types of muscle damage by activating endogenous RARγ signals [39]. However, as an important nutrient and a substrate of RA, the effect of VitA on muscle injury repair and its molecular mechanism are still unclear. Therefore, this experiment used VitA as a therapeutic drug. In this study, hematoxylin and eosin staining showed that the diameter of new muscle fibers in the VitA treatment group was higher than that in the control group. VitA shortened the time of muscle injury repair in mice, increased the diameter and number of new muscle fibers, and accelerated the process of muscle injury repair in mice. Western blotting results showed that VitA up-regulated the expression levels of MyoG and Desmin in mouse muscles, indicating that VitA could affect the repair process of muscle injury by promoting cell differentiation. The expression of RARα in the VitA treatment group was higher than that in the control group at each stage of injury repair. Therefore, it is speculated that VitA may promote muscle damage repair by metabolizing to RA and binding to its nuclear receptor RARα.

In summary, VitA promotes C2C12 cell differentiation through the enhancement of RARα expression and its translocation into nucleus. Additionally, it accelerates muscle repair in mice after injury. Determining the pathway of VitA action will provide a theoretical basis for studying the role of VitA in muscle development and developing economic animal nutrition supplements and muscle disease treatment drugs.

## 5. Conclusions

After VIitA is metabolized into retinoic acid in cells, it promotes the differentiation of C2C12 cells by up-regulating the expression and nuclear translocation of retinoic acid receptor protein RARα.Moreover, VitA treatment fosters augmented muscle fiber count and diameter, thereby expediting post-injury muscle recovery in mice. The mechanism of VitA promoting skeletal muscle injury and repair by promoting muscle differentiation can provide a scientific basis for the development of animal nutrition supplements and the treatment of muscle-related diseases.

## Data Availability

The data presented in this study are available in the article.
